# Psychological symptoms in individuals with Spinal Muscular Atrophy (SMA) and their caregivers – results from a nation-wide study in Germany

**DOI:** 10.1186/s13023-026-04558-1

**Published:** 2026-08-26

**Authors:** Justine Hussong, Berenike Leibrock, Tabea Huelle, Hannah Mattheus, Erik Landfeldt, Simone Thiele, Maggie C. Walter, Michael Zemlin, Eva Moehler, Ulrich Dillmann, Marina Flotats‑Bastardas

**Affiliations:** 1 Department of Child and Adolescent Psychiatry, Saarland University, 66421 Homburg, Germany; 2 Department of Medicine, University of Saarland, Saarbruecken, Germany; 3 Clinical Psychology and Psychotherapy, Saarland University, Saarland, Germany; 4 IQVIA, Stockholm, Sweden; 5 Friedrich Baur Institute at the Department of Neurology, LMU University Hospital, LMU, Munich, Germany; 6 Department of General Pediatrics and Neonatology, Saarland University, Homburg, Germany; 7 Department of Neurology, Saarland University, Homburg, Germany; 8 Department of General Pediatrics and Neonatology, Division of Neuropaediatrics, Saarland University, Homburg, Germany

**Keywords:** Spinal Muscular Atrophy (SMA), Psychological symptoms, Caregivers, Internalizing symptoms, Mental health, Psychosocial care

## Abstract

**Background:**

This study investigates the prevalence and associations of psychological symptoms in individuals with Spinal Muscular Atrophy (SMA) and their caregivers, utilizing data from a cross-sectional, observational assessment conducted in Germany. Participants were recruited through the national German SMA registry (June – September 2021), and psychological symptoms were assessed using validated measures such as the Strengths and Difficulties Questionnaire (SDQ) in children with SMA (*n* = 21) and the German Mini-Symptom-Checklist (Mini-SCL) in adults with SMA (*n* = 82) and caregivers (*n* = 67).

**Results:**

Results indicate that children with SMA exhibit lower rates of psychological symptoms compared to adults (9.5% vs. 13.4%), with internalizing symptoms (emotional problems, depression, anxiety) being the most prevalent in both age groups. Caregivers also demonstrate psychological symptoms in 14.9%, particularly those of individuals with SMA type 1. Symptom rates did not differ between groups with different motor function level. Significant correlations between caregiver and patient psychological symptoms were observed, while pharmacological treatment showed no significant impact on symptom rates.

**Conclusions:**

Psychological symptoms are common in individuals with SMA and their caregivers, particularly internalizing symptoms, underscoring the importance of routine psychological screening. Additionally, the correlation of patient and caregiver symptoms highlight the interplay between the mental health of both. These findings underscore the importance of integrating psychosocial care for both individuals with SMA and their caregivers to alleviate stress and promote well-being.

**Trial registration:**

German clinical trial register (DRKS), DRKS00022876. Registered 19 October 2020.

**Supplementary information:**

The online version contains supplementary material available at https://doi.org/10.1186/s13023-026-04558-1.

## Background

Persons with rare diseases or chronic illnesses have a higher risk of developing mental health problems, especially anxiety and depression [1, 2]. Affected children and adolescents often develop symptoms due to maladjustment, school problems, or reduced social participation. Their parents and caregivers usually report higher perceived stress and lower quality of life which increase the risk of own psychological problems [1, 3]. A recent systematic analysis of the psychosocial situation of caregivers of children with spinal muscular atrophy (SMA) identified multiple stressors that may cause mental health problems in the long term, e.g. parental distress or insufficient supportive care [4].

The present paper focuses on psychological symptoms in persons with SMA and their caregivers, as little is known about the associations between symptoms of caregiver and patient, as well as about the impact of motor function and new pharmacological treatments on mental health of both.

### Spinal muscular atrophy

Spinal muscular atrophy (SMA) is a rare autosomal recessive disease causing degeneration and loss of motor neurons of the spinal cord leading to a progressive impairment of motor skills, physical disability and in severe cases to a reduced life expectancy [5]. Prevalence of SMA in Europe is about 13–14 per 100,000 live births [6] and is caused by mutations in the survival motor neuron (*SMN1*) gene [5].

The severity of SMA is variable and can be classified into different phenotypes according to age of symptom onset and highest achieved motor function [5]. In severe SMA type 1 (SMA1) symptoms begin in the first half year of life, children never learn to sit and have a reduced life expectancy (<2 years) when untreated. Persons affected by SMA type 2 (SMA2) show motor regression before the age of 18 months. Sitting is the highest achievable motor function but can be lost over the time. Without therapy life expectancy is reduced. In SMA type 3 (SMA3), children learn to walk on his own but muscle weakness appear beyond the age of 18 months, and without therapy some of them are over the time wheel chair bound. Individuals with SMA type 4 (SMA4) show muscle weakness in the adulthood but they don’t lose the ability to walk. This existence and frequency of this type is controversial, as in many patients, first mild (less disabling) symptoms may appear in young adulthood [7].

In the last years, new gene therapy treatments became available (i.e., nusinersen, onasemnogene abeparvovec, and risdiplam) leading to major improvements of the motor function and thus improving dramatically the prognosis of SMA [7]. Therefore the prevalence of SMA is increasing and new phenotypes are appearing, thus a classification by the current level of current motor function (non-sitters, sitters, walkers) help clinicians to adjust and allow the appropriate medical care [8].

### Mental health problems in individuals with SMA and their caregivers

Psychological symptoms in children with SMA are reported in 12%-40% [9–11]. Externalizing symptoms (e.g. attention-deficit, hyperactivity, aggressive behavior) are rather uncommon (in 0–2%) and not higher than in control groups, whereas slightly higher rates (in 7%-19%) of internalizing symptoms (e.g. depression, anxiety) are reported [9, 10, 12], except for one Chinese study that found even higher rates of anxiety (40%) and depression (25%) in children with SMA [11].

Less is known about psychological symptoms in adults with SMA. In a symptom list assessed in 70 adult German patients with SMA2+3 compared to 59 healthy controls, frequency of self-reported anxiety (17%), loss of interest (9%), concentration difficulties (20%), and insomnia (31%) was not significantly increased in SMA patients and did not differ between SMA2 and SMA3 [13]. Further evaluations from Germany and Malaysia reported anxiety and depressive symptoms in 29–38% of adults with SMA [14, 15].

Regarding caregivers, studies report clinical depression symptoms in 21–36%, and clinical anxiety symptoms in 26–76% [14, 16–19]. Parental depression symptoms were associated with higher children’s and parental age, patient living at home and medical treatment (with nursinersen), and absence of rehabilitation management was associated with caregivers’ anxiety symptoms [18, 19]. The association of parental emotional symptoms and SMA type were inconsistent [16, 19].

The effect of modern SMA medical treatment on psychological symptoms in patients is scarcely examined so far. One study evaluated 26 patients (14 adults, 12 children) before and after nursinersen treatment and showed no change of depressive symptoms after 6 months of treatment [15]. Another study from Turkey found even higher depression scores in caregivers of treated SMA children compared to untreated children [19].

The aim of our study was to explore psychological symptoms in children and adults with SMA, as well as in their caregivers.

## Methods

The present study is part of a cross-sectional, observational assessment of health-related quality of life (HRQoL) and psychological symptoms [20, 21] among individuals with SMA and their caregivers. Detailed information on the psychosocial background of the families, as well as treatment-related variables have been comprehensively described in our previous publication by Leibrock et al. [20]. Results of HRQoL among children and adults with SMA, measured using different tools (e.g. KIDSCREEN-27, KINDL, the PedsQL, EQ-5D-5L, and the Health Utilities Index), have been published by Landfeldt et al. [21]. The present study focuses on psychological symptoms in the sample.

### Sample

Patients were recruited trough the national German SMA registry (http://www.sma-register.dewww.sma-register.de), which is part of the ‘Translational Research in Europe for the Assessment and Treatment of Neuromuscular Disease’ (TREAT-NMD) network. TREAT-NMD aims to facilitate recruitment of patients to neuromuscular research, in order to raise awareness and improve treatment methods. The enrollment in the SMA registry is voluntary and free of charge. To be recruited for the present study, participants had to meet all of the following criteria: (1) genetically confirmed diagnosis of SMA, (2) ≥4 years of age, and (3) currently residing in Germany. A study invitation and information sheet was sent via email by the SMA registry to all listed individuals meeting the study criteria. In children (participants < 18 years), parents/caregivers were contacted. After provided informed consent, patients and one of their caregivers were invited to complete a questionnaire administered online via the study website http://www.soscisurvey.de. To be considered eligible to participate in this study, caregivers had to meet all of the following criteria: (1) involved in and/or responsible for the informal care of the patient with SMA, (2) cares for the patient with SMA in a non-professional capacity, and (3) has up-to-date knowledge about the patient’s day-to-day needs and current situation. The study was approved by the local Ethics Committee of the Saarland Medical Association (February 4, 2020, protocol no. 09/20) and was registered at the German clinical trial register (DRKS00022876).

### Materials and collected data

The online questionnaire included a *general part about demographic and clinical characteristics* which is described in detail by Leibrock et al. [20]. The data collection took place between June and September 2021.

To assess psychological symptoms in children and adolescents with SMA, the parental version of the *Strengths and Difficulties Questionnaire (SDQ)* (German version) was completed by parents/caregivers in children aged 3 years or older, and as self-report in children from the age of 12 years onwards. The SDQ is an internationally validated, standardized screening questionnaire for 3–17 year old children and adolescents consisting of 25 items about psychological and behavioral symptoms, each scored on a three-point scale (not true (0) – somewhat true (1) – certainly true(2)) [22]. Five subscales can be calculated (each from 5 items): emotional symptoms (5 items), conduct problems (5 items), hyperactivity (5 items), peer problems (5 items) and prosocial behavior (5 items). The total problem behavior score (TPBS) is calculated as a sum of the first four scale scores except ‘prosocial behavior’. According to German norms, subscale scores and the TBPS were divided into clinical (>90th percentile), borderline (80–90th percentile), and average (<80th percentile) cut-offs [23, 24]. The clinical cut-off at the 90th percentile was used as identification of clinically relevant mental health symptoms.

To assess psychological symptoms in adult individuals with SMA and in parents/caregivers, the German *Mini-Symptom-Checklist (Mini-SCL)* self-report was completed [25]. The Mini-SCL, also known as Brief Symptom Inventory (BSI-18) is a validated and reliable short form of the Symptom Checklist (SCL-90-R). Three scales (somatization, depression and anxiety) assessed by 6 items each, as well as the Global Severity Index (GSI) based on all 18 items can be calculated. The 18 items refer to current psychological symptoms and are rated on a 5-point Likert-scale ranging from 0 (not at all) to 4 (extremely). Psychometric properties of the Mini-SCL are satisfactory or good for all subscales and the GSI. According to German norms, the 93rd percentile (~T = 65) is used as a cut-off for clinically relevant symptoms [25]. The parent/caregiver also filled in the *Mini-SCL self-version* in order to assess their own mental health symptoms.

### Statistical analysis

Statistical calculations were performed with IBM SPSS Statistics Version 25. Descriptive data was reported by relative and absolute frequencies for non-parametric variables and by means and standard deviation (SD) for parametric data. Group differences for nominal data were calculated by Chi^2^-tests and Fisher’s exact tests. Correlations were calculated by Spearman correlations. Results are considered as significant at a p-value < 0.05.

## Results

Out of 742 patients enrolled in the SMA patient registry, 513 met the inclusion criteria, and 117 agreed to participate in the study. Completed data on mental health problems were available for *n* = 103 (*n* = 21 children and *n* = 82 adults), in *n* = 14 cases questionnaires were incomplete.For *n* = 67 cases (of *n* = 103), additional caregiver data were available. Descriptive data of the sample (21 children, 82 adults) is provided in Table 1. Participants were classified based on SMA subtype (i.e., SMA1, 2, and 3) as well as according to current best motor function of the lower limb (i.e., non-sitter, sitter, and walker).

**Table 1:** Descriptive data by SMA subtype (sex, age, motor function, medication intake)

	Total	Children (<18 years)	Adults
	N = 103	N = 21	N = 82
*Mean age in years (SD)*	*35.8 (19.0)*	*9.7 (4.5)*	*42.5 (15.1)*
Sex distribution in % (n)			
* Male*	47.6 (49)	47.6 (10)	47.6 (39)
* Female*	51.5 (53)	52.4 (11)	51.2 (42)
* Other*	1.0 (1)	-	1.2 (1)
Subtype in % (n)			
* SMA1*	6.8 (7)	19.0 (4)	3.7 (3)
* SMA2*	35.9 (37)	42.9 (9)	34.1 (28)
* SMA3*	57.3 (59)	38.1 (8)	62.2 (51)
Best current motor function in % (n)			
* Non-sitter*	9.7 (10)	14.3 (3)	8.5 (7)
* Sitter*	64.1 (66)	52.4 (11)	67.1 (55)
* Walker*	26.2 (27)	33.3 (7)	24.4 (20)
Medication intake in % (n)			
* None*	22.3 (23)	9.5 (2)	25.6 (21)
* Nursinersen*	55.3 (57)	71.4 (15)	51.2 (42)
* Risdiplam*	22.3 (23)	19.0 (4)	23.2 (19)

### Clinically relevant mental health symptoms in children, adults and caregivers

**Fig. 1:**
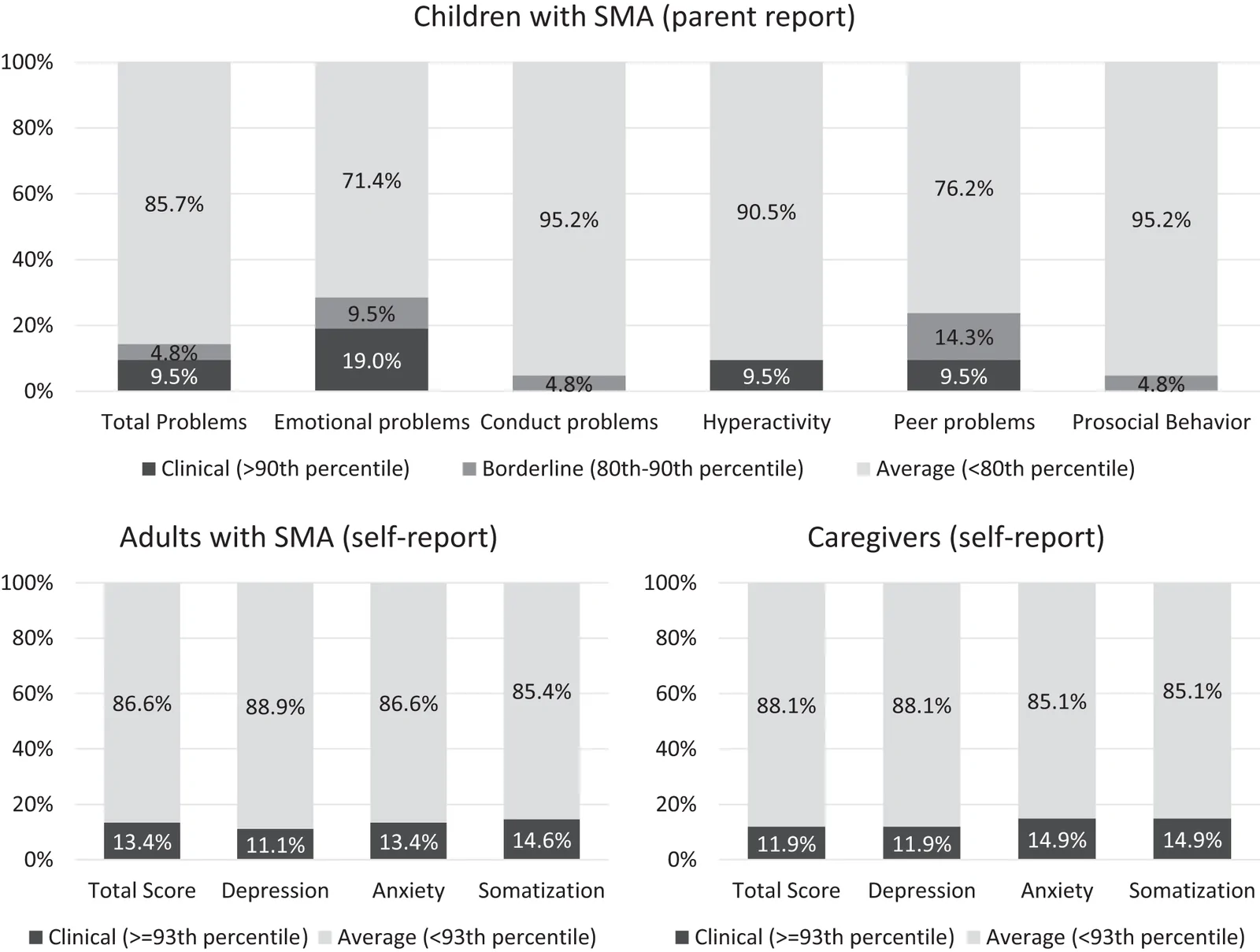
Clinically relevant psychological symptoms in children, adults with SMA and caregivers/close relatives (self-report)

Figure 1 presents rates of psychological symptoms in the clinical and average range in children, adults with SMA and caregivers focusing on the assessed symptom scales and Table 2 shows only clinically relevant psychological symptoms in children and adults with SMA in the total sample, differed by SMA subtypes and motor function. In children (*n* = 21), 10% show relevant mental health problems being emotional problems (19%), hyperactivity (10%) and peer problems (10%) the most common one recorded by SDQ parental report. Regarding SMA types, peer problems were mainly reported in SMA1 (50%) and emotional problems were highest in SMA3 (38%). Regarding motor function, emotional problems were seen in non-sitters and walkers (33% and 29% respectively). Self-reports were available for *n* = 7 children and no symptoms in the clinical range were described. Only one child reported borderline (80^th^-90th percentile) symptoms of hyperactivity, and another one reported lower prosocial behavior in the borderline range. Mean scores (SDs) of the SDQ scales (self- and parental report) are summarized in the *additional file* *1*.

**Table 2:** Clinically relevant psychological symptoms in children (proxy report), adults with SMA and caregivers/close relatives (self-report)

	Total	SMA subtypes			**P**^**c**^	Lower limb motor function			**P**^**c**^
		SMA1	SMA2	**SMA3**^**e**^		Non-Sitters	Sitters	Walkers	
**Children with SMA –***Parent reported SDQ – % in the clinical range (n)*^*a*^	*n = 21*	*n = 4*	*n = 9*	*n = 8*		*n = 3*	*n = 11*	*n = 7*	
Emotional problems	19.0 (4)	0 (0)	11.1 (1)	37.5 (3)	n.s.	33.3 (1)	9.1 (1)	28.6 (2)	n.s.
Conduct problems	0 (0)	0 (0)	0 (0)	0 (0)	-	0 (0)	0 (0)	0 (0)	-
Hyperactivity	9.5 (2)	0 (0)	11.1 (1)	12.5 (1)	n.s.	0 (0)	9.1 (1)	14.3 (1)	n.s.
Peer Problems	9.5 (2)	50.0 (2)	0 (0)	0 (0)	0.029*	0 (0)	18.2 (2)	0 (0)	n.s.
Prosocial Behavior	0 (0)	0 (0)	0 (0)	0 (0)	-	0 (0)	0 (0)	0 (0)	-
Total	9.5 (2)	0 (0)	11.1 (1)	12.5 (1)	n.s.	33.3 (1)	0 (0)	14.3 (1)	n.s.
**Adults with SMA –***Self-reported Mini-SCL – % in the clinical range (n)*^*a*^	*n = 82*	*n = 3*	*n = 28*	*n = 51*		*n = 7*	*n = 55*	*n = 20*	
Depression	11.0 (9)	0 (0)	3.6 (1)	15.7 (8)	n.s.	14.3 (1)	9.1 (5)	15.0 (3)	n.s.
Anxiety	14.6 (12)	0 (0)	3.6 (1)	21.6 (11)	n.s.	28.6 (2)	12.7 (7)	15.0 (3)	n.s.
Somatization	14.6 (12)	33.3 (1)	10.7 (3)	15.7 (8)	n.s.	28.6 (2)	14.5 (8)	10.0 (2)	n.s.
Total	13.4 (11)	0 (0)	3.6 (1)	19.6 (10)	n.s.	28.6 (2)	9.1 (5)	20.0 (4)	n.s.
**Caregivers –***Self-reported Mini-SCL – % in the clinical range (n)*^*b*^**in % (n)**^**b**^	*n = 67*	*n = 6*	*n = 25*	*n = 36*		*n = 10*	*n = 40*	*n = 17*	
Depression	11.9 (8)	33.3 (2)	0 (0)	16.7 (6)	0.021* (1 > 2)	0 (0)	15.0 (6)	11.8 (2)	n.s.
Anxiety	14.9 (10)	50.0 (3)	4.0 (1)	16.7 (6)	0.022* (1 > 2)	10.0 (1)	15.0 (6)	17.6 (3)	n.s.
Somatization	14.9 (10)	16.7 (1)	4.0 (1)	22.2 (8)	n.s.	20.0 (2)	12.5 (5)	17.6 (3)	n.s.
Total	14.9 (10)	16.7 (1)	4.0 (1)	22.2 (8)	n.s.	10.0 (1)	15.0 (6)	17.6 (3)	n.s.

^a^ based on parental SDQ (Strengths and Difficulties Questionnaire) scores > 90th percentile

^b^ based on self-reported Mini-SCL (Symptom Checklist) scores > *T* = 65 (~93rd percentile)

^c^ Fisher’s Exact test if not otherwise specified

^d^ Depression scale reports available for *n* = 81 (*n* = 27 in type II, *n* = 54 in non-sitters)

^e^ SMA3 also included one patient with SMA4

**Fig. 2:**
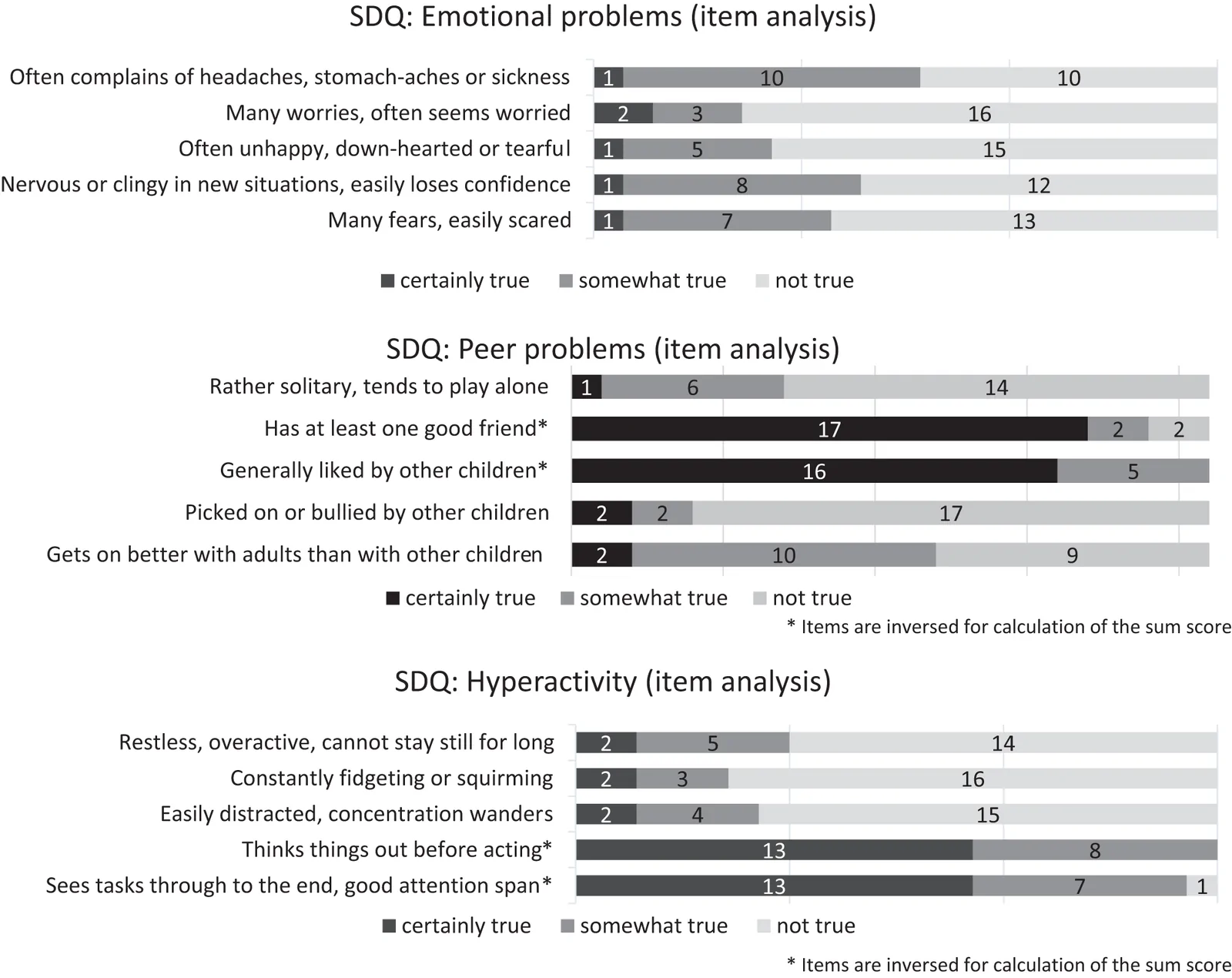
Item analysis of SDQ scales ‘Emotional problems’, ‘Peer problems’ and ‘Hyperactivity’(proxy-report)

Figure 2 presents an item analysis of the three SDQ scales ‘emotional’, ‘peer problems’ and ‘hyperactivity’ in which symptoms were reported most often in the clinical range. The most frequent reported symptoms in the emotional problems scale were ‘often complains of headaches, stomach-aches or sickness’ (in 11/21 children) and ‘nervous or clingy in new situations, easily loses confidence’ (in 9/21 children). Most often reported items in the peer problems scale were ‘gets on better with adults than with other children’ (in 12/21 children) and ‘rather solitary, tends to play alone’ (in 7/21 children). Regarding the hyperactivity scale, most frequent reported symptoms are ‘doesn’t think things out before acting’ and ‘doesn’t see tasks through to the end’ (in 8/21 children each).

In adults (*n* = 82) clinically relevant psychological symptoms recorded by Mini-SCL self-report are reported by 13% of adults (Fig. 1 and Table 2). Adults with SMA3 had the highest rates of anxiety (22%) and depression (16%), whereas somatization was reported in all SMA types. Regarding motor function, symptom rates were highest among non-sitters (29%) mainly because of anxiety and somatization, followed from walkers (20%) mainly because of depression and anxiety. However, no differences between SMA types or motor function reached statistical significance.

**Fig. 3:**
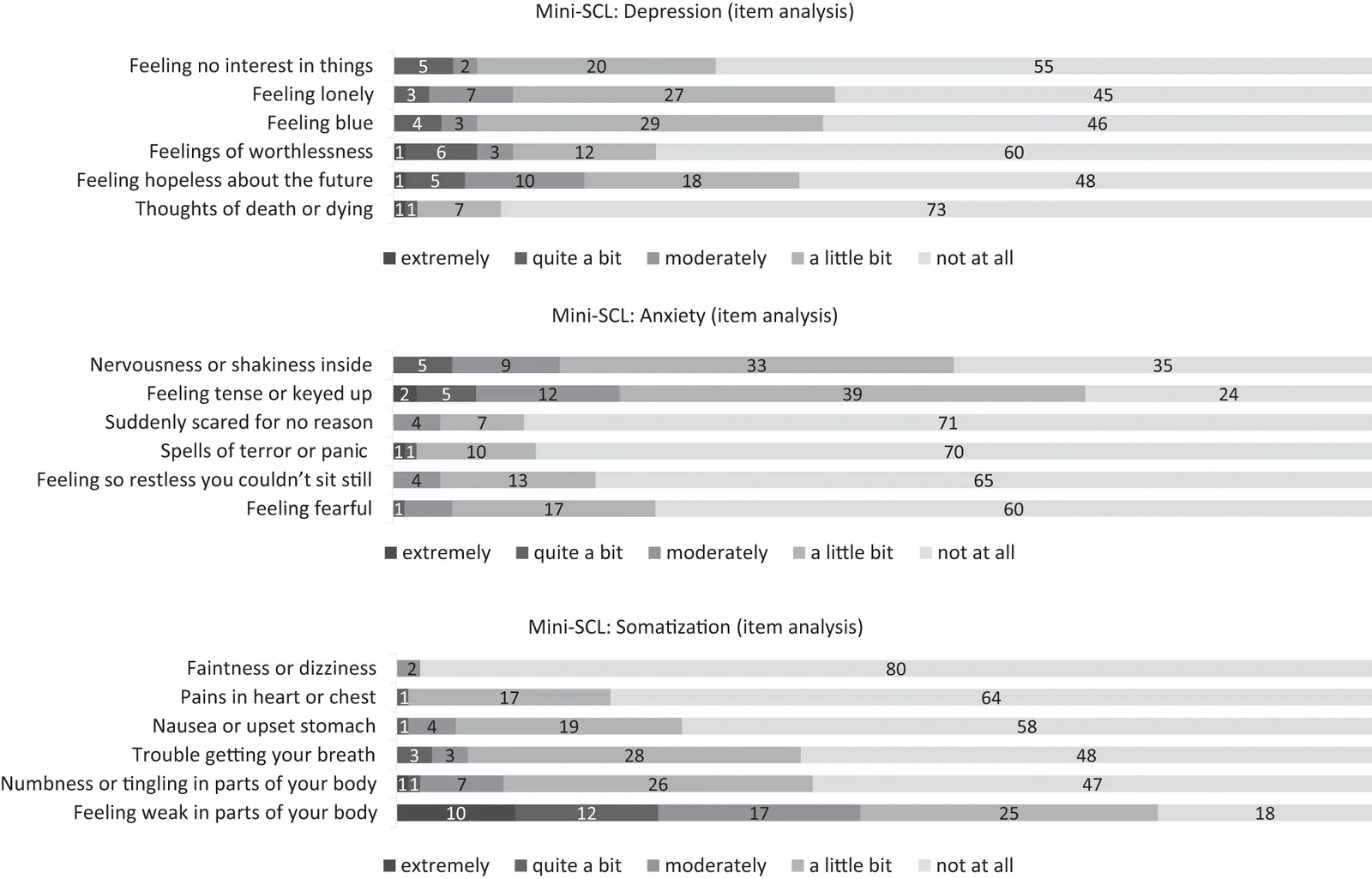
Item analysis of Mini-SCL scales ‘Depression’, ‘Anxiety’ and ‘Somatization’in adults with SMA (self-report)

An item analysis of the ‘depression’, ‘anxiety’ and ‘somatization’ scales of the self-report of adults with SMA is shown in Fig. 3. The most frequent depression symptoms were ‘feeling lonely’ and ‘feeling hopeless about the future’. The most frequent anxiety symptoms were ‘nervousness or shakiness inside’ and ‘feeling tense or keyed up’. Regarding somatization, the most frequent symptoms were ‘feeling weak in parts of the body’ and ‘numbness or tingling in parts of the body’.

Mental health symptoms of caregivers *n* = 67 (mostly parents of 21 children, 46 adults) are outlined in Fig. 1 and Table 2. Parents reported own clinically relevant psychological symptoms in 15% of cases. Regarding SMA type, 22% of caregivers of individuals with SMA3 complain about psychological symptoms, mainly because of somatization whereas 17% of caregivers of individuals with SMA1 mainly complain about anxiety and depression. Regarding motor function, symptom rates were highest among caregivers of walkers (18%) because of anxiety and somatization, followed from sitters (15%) because of anxiety and depression and non-sitters (10%) because of somatization. An item analysis of the ‘depression’, ‘anxiety’ and ‘somatization’ scales of the self-report of caregivers can be found in the *additional file* *3*.

### Association of patient and caregiver mental health symptoms

Parental symptoms of depression are significantly associated with their children’s emotional problems, peer problems and the total score. Parental anxiety was correlated to children’s conduct problems. In adults with SMA, nearly all scales were significantly correlated to those in their parents/caregivers, revealing a strong association between the symptoms of patient and caregiver. Significant correlations between mental health symptoms of SMA patients and of their parents and caregivers are shown in the *additional file* *2*.

### Association of mental health symptoms and pharmacological treatment

Table 3 reports mental health symptoms of children, adults and caregivers depending on their current pharmacological treatment. Overall, there is no statistical difference in the extent of psychological symptoms between patients with no medication, those treated with nursinersen and those treated with risdiplam. Regarding children with SMA, clinical scores were only reported in individuals treated with medication (*n* = 19) in contrast to those with no medication (*n* = 2). In adults with SMA (medication *n* = 61, no medication *n* = 21) clinical scores for somatization and anxiety where higher in those individuals without medication (24% and 19% vs 11% and 13%). The most frequent reported symptoms were somatization, with the highest rates in persons with no medication (23.8%) and those treated with risdiplam (21.1%), in contrast to lower symptom rates (7.1%) in the group treated with nursinersen. Mental health of caregivers does not differ between the medication groups, as well. The most frequent reported symptoms are somatization in the risdiplam group (21.4%), anxiety and depression in the nursinersen group (17.1% each), as well as anxiety and total symptoms in the ‘no medication’ group (16.7% each).

**Table 3:** Association of mental health symptoms (children, adults and caregivers) and pharmacological treatment^c^

	No medication	Medication	Nursinersen	Risdiplam
**Children with SMA –***Parent reported SDQ – % in the clinical range (n)*^*a*^	*N = 2*	*N = 19*	*N = 15*	*N = 4*
Emotional problems	0.0 (0)	21.1 (4)	13.3 (2)	50.0 (2)
Conduct problems	0.0 (0)	0.0 (0)	0.0 (0)	0.0 (0)
Hyperactivity	0.0 (0)	10.5 (2)	13.3 (2)	0.0 (0)
Peer Problems	50.0 (1)	5.3 (1)	6.7 (1)	0.0 (0)
Prosocial Behavior	0.0 (0)	0.0 (0)	0.0 (0)	0.0 (0)
Total	0.0 (0)	10.5 (2)	6.7 (1)	25.0 (1)
**Adults with SMA –***Self-reported Mini-SCL – % in the clinical range (n)*^*b*^	*N = 21*	*N = 61*	*N = 42*	*N = 19*
Depression	9.5 (2)	11.5 (7)	11.9 (5)	10.5 (2)
Anxiety	19.0 (4)	13.1 (8)	11.9 (5)	15.8 (3)
Somatization	23.8 (5)	11.5 (7)	7.1 (3)	21.1 (4)
Total	9.5 (2)	14.8 (9)	14.3 (6)	15.8 (3)
**Caregivers –***Self-reported Mini-SCL – % in the clinical range (n)*^*b*^	*N = 12*	*N = 55*	*N = 41*	*N = 14*
Depression	8.3 (1)	12.7 (7)	17.1 (7)	0.0 (0)
Anxiety	16.7 (2)	14.5 (8)	17.1 (7)	7.1 (1)
Somatization	8.3 (1)	16.4 (9)	14.6 (6)	21.4 (3)
Total	16.7 (2)	14.5 (8)	17.1 (7)	7.1 (1)

^a^ based on parental SDQ (Strengths and Difficulties Questionnaire) scores > 90th percentile

^b^ based on self-reported Mini-SCL (Symptom Checklist) scores > *T* = 65 (~93rd percentile)

^c^ Fisher’s Exact test revealed no significant differences between medication and no medication group

## Discussion

To our knowledge, this is the first detailed description of psychological symptoms assessed by standardized questionnaires and self- and proxy-reports in SMA children, adults and their caregivers. Our data include all SMA subtypes, levels of motor function and pharmacological treatment statuses. Children were less affected by psychological symptoms than adults with SMA, with internalizing symptoms being the most common symptoms reported in both groups. Internalizing symptoms were as well present in caregivers, especially in those of persons with SMA1. Caregiver and patient psychological symptoms were strongly associated, whereas pharmacological treatment seem to not affect symptom rates.

### Psychological symptoms in children

Psychological symptoms in children ranged from 0% in self-reports to emotional problems in 19% rated by parents. Children and adolescents rated themselves as less impaired as their parents did. The self-ratings must be interpreted cautiously, because only 7 adolescents (age range 12–16 years) completed the questionnaires. Otherwise, in parental rating, reported symptoms may have been mixed with parents’ own worries about their child and perceived stress which could overestimate symptom rates.

Psychological symptoms in children were assessed by self-report only by Yao et al. [11] before, who found much higher rates of anxiety (40%) and depression (25%), being unusually high compared to other studies on children with SMA [9, 10].

The low rates of psychological symptoms (especially in children) in our study may result from the access to medical care and social support systems, which is fairly good for children (but not quite for SMA adults) in Germany and allows a sufficient participation in social and public life [20]. Leibrock et al. present socioeconomic characteristics and details on medical care of the present sample, which are included to the discussion of potential associations [20]. In our sample, more children with SMA1 (21% vs. 8%) were included, as many more children received pharmacological treatment (92% vs. 5%) than in the Chinese study population [11]. Also, as reported by Leibrock et al., all children in our sample received supportive therapy, especially physiotherapy (vs. 54% of Chinese children with ‘rehabilitation exercise’) and all German children visited an appropriate school (vs. traditional schooling in 69% and academic delay in 55% in Chinese children) [11, 20]. The Chinese colleagues also found a specific association between psychological symptoms and the less available social and medical support [11], which is in line with research outcomes stating clearly that social participation, e.g. school visits, and an adequate medical care are protective factors for mental health [26].

In line with other publications, internalizing symptoms (as anxiety, depression) were more common than externalizing problems (e.g. aggressive behavior, hyperactivity, ADHD symptoms) in the present sample [9, 10, 12]. All in all, the rates of internalizing symptoms are only slightly higher than known in the German normative population (~10–15% [27]); and could be symptoms of so-called ‘pediatric medical traumatic stress’ that occurs in patients and parents after learning about a serious diagnosis or invasive medical treatments [28].

Further, most SMA patients have good cognitive and adaptive abilities [9, 12] are further protective factors that promote mental well-being, as e.g. genetic syndromes that are associated with intellectual disability reveal much higher rates of concomitant psychiatric diagnoses than individuals with SMA [29].

There was only one significant difference between SMA types, for peer problems were mainly reported in SMA1 (in 50%), but only by parental rating. Regarding the item analysis of the peer problems scale, it was reported that affected children ‘get on better with adults than with other children’ and ‘rather solitary, tend to play alone’, which could reflect a social exclusion and missing social participation opportunities in children with SMA. Peer problems were not assessed in children with SMA before, but were also higher in a sample of children with rare diseases [2]. Children with SMA1 have the most severe type of disease, with extremely limiting physical impairments, e.g. including the need of ventilatory support, which of course may lead to social problems or difficulties in interacting with other children. Otherwise, with the new pharmacological treatments, these children have a much better outcome and a longer survival prognosis, so that increasing and improving the opportunities to social participation in these patients is a relevant clinical implication for the future.

Regarding the level of motor function, our data showed no significant differences in mental health symptoms between the groups. The association of mental health and motor function of the lower limbs was not assessed before, so we may assume that motor function may be independent from psychological symptoms. Hopefully, future studies with larger sample sizes will reveal more information to that topic.

### Psychological symptoms in adults

Adults with SMA report psychological symptoms in 11% (depression) to 15% (somatization). These symptom rates are slightly higher than in children but lower than in the few studies known on adults with SMA [13–15]. However, only Mix et al. used a validated instrument for assessing depressive symptoms [15], others worked e.g. with non-standardized symptom lists and qualitative research, which could have overestimated symptom rates [13, 14]. Günther et al. found symptoms of e.g. anxiety in 17%, loss of interest in 9%, and insomnia in 31%, but all rates were not higher than in the healthy control group [13]. These symptoms can indicate a psychiatric disorder, but one can assume that not all of these participants would receive a manifest diagnosis e.g. of depression. We found neither differences between SMA types nor between groups of lower limb motor level. Former studies did not find SMA type differences as well [13], but psychological symptoms were associated with social participation and physical function [15, 30]. Regarding the item analysis of the depression and anxiety scale in our adult sample, the most common symptoms reported can be connected to physical/somatic complaints (‘nervousness/shakiness’, ‘feeling tense or keyed up’) or to a deficient social participation (‘feeling lonely’, ‘feeling hopeless’).

As seen in children with SMA, a functioning social support system and access to medical care are relevant factors for participation in daily life and mental well-being. Qualitative research from Australia on healthcare experiences of SMA patients during transition into adulthood reveals some distressing problems, e.g. that adult healthcare services is perceived as less structured and of lower quality, the reliance on others for assistance with basic needs, or social stigmatization. Further, participants described feelings of sadness in reaction to decreasing functional capability and autonomy [31]. These aspects play a major role in the life of adults with SMA and may explain well the increased rates of psychological symptoms.

The small sample sizes may be an explanation for the missing differences between groups of motor function, as the rates are highest among non-sitters (somatization and anxiety symptoms in 28.6%). The results could have missed statistical significance, as only 7 adults were included in that subgroup.

These findings from Mix et al. show that depressive symptoms correlated significantly with better physical function and remained unaffected after treatment [15]. As in our sample only anxiety and somatization symptoms were higher in non-sitters, future studies should not only focus depression, but also include other psychiatric disorders, e.g. anxiety disorders with their different aspects. One can speculate that a decreased physical ability may cause social phobia or lead to a generalized anxiety characterized by concerns or fear towards future and progress of the disease. The Mini-SCL subscale ‘somatization’ includes questions about ‘breathing difficulties’ or ‘feelings of weakness in body parts’, which could be confounded with symptoms of severe SMA and therefore could have caused higher ratings in that scale.

### Psychological symptoms in caregivers

Mental health symptoms of caregivers were reported in 15% of cases, notably anxiety and somatization in 15%. The rates of the total sample are slightly lower than in other studies on SMA caregivers, which reported depression symptoms in 21–36%, and anxiety symptoms in 26–76% of caregivers [14, 16–19]. With focus on SMA types, we see significantly more psychological problems in parents of patients with SMA1, showing similarly high rates than former studies (33% depression, 50% anxiety). Caregivers of individuals with this severe type of SMA seem to have the highest risk of developing mental health problems. These may be caused by higher stress and involvement with the care and support of the affected person. A study from Saudi Arabia found the same association in caregivers of SMA1 patients [16], and also found higher hospitalization and needs for mechanical support (e.g. ventilatory) in these patients. In our sample, all of SMA1 individuals needed ventilatory support, 80% needed food intake support, all needed medical equipment (e.g. orthoses, wheelchair) and all received any kind of supportive therapy [20].

In a study on 96 children with SMA, parental stress was significantly higher in parents of children with SMA1+2 than in those with SMA3. Parental stress was significantly correlated to psychological symptoms and could be predicted by social support and number of children in the family [32]. A newer study on a Dutch sample revealed a significant association between emotional well-being of SMA caregivers and participation in social and leisure activities [17], which explains very well the increased psychological symptoms in caregivers, especially of persons with SMA1. One can imagine that especially caregivers of the SMA1 group invest much more time in the care and organization of support which can easily lead to higher perceived stress, mental overload, less time for other social activities or self-care.

The level of achieved motor function seems to not affect psychological symptoms of caregivers. That means that other factors must have a higher impact on mental health on caregivers, e.g. the access to support systems or further assistance.

### Association of symptoms between caregiver and individual with SMA

The significant correlations between caregiver and patient psychological symptoms confirm the strong association regarding emotional well-being of the whole family system, not only in young patients, but in adults, as well. Correlations between parental and child psychological symptoms were assessed only in one Chinese study on children with SMA, so far, reporting a strong connection of depression and anxiety, too [11].

Our results on children reveal associations of psychological symptoms that have not been assessed before, e.g. that parental symptoms of depression are significantly associated with their children’s emotional and peer problems; as well as that parental anxiety was correlated to children’s conduct problems.

As symptoms were assessed only in a cross-sectional study design, we can only speculate about causal attributions, but several ways of associations are possible. The diagnosis of a rare and chronic illness of the child, as well as the following responsibilities and care duties can cause traumatic stress in child and parents, which increases the risk of mental health problems in both [28]. Parental stress is associated to behavioral problems of the child in SMA, as well to social support and level of disability [32].

The association between parental anxiety and conduct problems of the child may also be explained bi-directionally. Conduct problems of the child (e.g. aggressive behavior) can cause anxiety of parents, as they worry about their future, fear social problems or exclusion of their child. Or otherwise, anxious parents could tend to overprotect their child, be less strict or show a more inconsequent parenting style, which may cause conduct problems. A systematic analysis of parent-child interaction in families with a chronic ill child supports these hypotheses. Parents of children with a chronic physical illness tended to be less positive and showed higher levels of responsiveness, but also of control and overprotection [33]. Future studies should include longitudinal analysis on psychological symptoms in caregivers and patients to provide more information about the directions of symptom associations.

In adults with SMA, nearly all scales were significantly correlated to those in their parents/caregivers, revealing a strong association between the symptoms, as well. To our knowledge, this connection has not been assessed in adults with SMA before. But it means that although ~70% of adults in our sample do not live with their parents anymore [20], there still exists a strong relationship regarding mental health in both. A Dutch study showed that rates of maternal depression (and anxiety) symptoms were not significantly higher in mothers of children than in mothers of adult patients [17], which supports our findings. One can state that the risk of psychological symptoms in caregivers does not decrease with age of the patient or because the patient moved out, but still impacts the social life and well-being of the caregiver.

The findings are in line with the implications of the systematic analysis on family needs in SMA stating that the needs of parents must be included in psychosocial care to prevent health problems due to stress [4]. Our results may add that this applies not only for parents of children with SMA, but equally to parents/caregivers of adult patients.

### Psychological symptoms and pharmacological treatment

The rates of psychological symptoms did not differ significantly between groups based on their current pharmacological treatment. In children, clinical scores were mainly reported in those treated with nursinersen, but one has to consider that the sample sizes of the other two groups (no medication *n* = 2; risdiplam *n* = 4) were too low to interpret these data. In adults, reported clinical symptoms were distributed similarly among the groups, with no specific trend observed. Mental health symptoms of caregivers did not differ between medication groups statistically. According to the small sample sizes, interpretation of rate differences should be undertaken with great caution. Studies with larger sample sizes or longitudinal data are needed to confirm these findings and to allow for more robust and reliable conclusions.

There are only a few other studies on this topic. Mix et al. evaluated 26 patients (14 adults, 12 children) before and after nursinersen treatment and revealed that depressive symptoms did not change after treatment [15]. An increase in physical function correlated even with more depressive symptoms. Ergenekon et al. showed a positive association between medical treatment and parental depression scores, as well [19]. The authors of these studies discuss that patients with poorer physical abilities have a better mental health, as they receive the SMA diagnosis at a young age and integrate it better into their self-concept which causes lower mental problems. The authors of the study on caregivers pose the question if a delayed treatment initiation may have contributed to higher parental depression rates.

The results imply that pharmacological treatment does not substantially improve psychological symptoms in either individuals with SMA or their caregivers, which is understandable, as such improvements would not be expected from pharmacological treatment alone. Manifest psychiatric disorders, e.g. a depression or anxiety disorder are usually caused by multiple biological, psychological and social factors. Further, treatment recommendations include a multimodal treatment approach [26]. Therefore, we cannot assume that the change in one factor (improvement of SMA symptoms) leads immediately to a better mental health outcome in patients or even their caregivers. In the long-term, the change of SMA prognosis due to medical treatment options may improve quality of life, lower perceived stress and parental burden; and this could decrease psychological symptoms as a secondary outcome after a certain period of time. This should be included into future research on long-term effects of pharmacological treatment in SMA.

### Strengths and limitations

Strengths of our study are the validated and standardized questionnaires allowing assessment of clinically relevant psychological symptoms in different age groups of persons with SMA, as well as their caregivers. Further, the sample size in adults with SMA was large enough to analyze differences between subtypes, levels of motor function and pharmacological treatment status regarding mental health. The combination of self- and proxy-reports, as well as of patient and caregiver reports revealed associations that were not assessed before.

Limitations are the recruitment via the nationwide registry that may include information biases and selection biases. As information was gained via an online questionnaire, biases due to incorrect reporting cannot be excluded. The sample size in children was too small to receive solid information on subgroup levels. Further, the cross-sectional design does not allow causal attributions.

### Clinical implications

The findings of the present study suggest several important clinical implications for the care of individuals with SMA and their families. As not all individuals are affected by mental health symptoms, we recommend a stepwise approach. First, routine psychological screening for both patients and primary caregivers could help to identify emerging mental health difficulties at an early stage. Integrating brief standardized screening tools into regular neuromuscular clinic visits may allow clinicians to detect symptoms of anxiety, depression, or caregiver burden and facilitate timely referrals.

Second, access to specialized psychological counseling or psychotherapy for both patients and caregivers may be beneficial. Such support could address disease-related stressors including uncertainty about disease progression, treatment decisions, caregiving demands, and the emotional impact of living with a chronic neuromuscular condition. Third, family-centered interventions may be especially valuable given the interconnected nature of psychological well-being observed in our data. Psychoeducational programs, family counseling, or structured support groups may help families develop adaptive coping strategies, improve communication about illness-related challenges, and strengthen resilience within the family system. Overall, our results support the integration of family-oriented psychosocial support structures into multidisciplinary SMA care. Embedding mental health professionals within neuromuscular care teams and facilitating access to psychological services may help address the substantial yet often unmet psychosocial needs of patients and their families.

## Conclusions

Our study represents a significant advancement in understanding the psychological symptomatology of individuals with SMA and their caregivers. Through the comprehensive assessment of standardized questionnaires and self- and proxy-reports, we have provided the first detailed description of psychological symptoms across SMA subtypes, levels of motor function, and in individuals receiving modern pharmacological treatment.

Our findings reveal that while children with SMA exhibit lower rates of psychological symptoms compared to adults, internalizing symptoms are predominant in both age groups, with caregivers of SMA1 individuals experiencing heightened psychological distress. Importantly, we have demonstrated a significant association between caregiver and patient psychological symptoms, underscoring the interdependence of mental health within SMA families. Furthermore, the influence of access to medical care and social support systems on the manifestation of psychological symptoms, particularly in adults with SMA, highlights the critical role of psychosocial interventions in promoting well-being in chronic-ill patients. The correlation between parental and patient psychopathology emphasizes the need for integrated psychosocial care tailored to both individuals with SMA and their caregivers to reduce stress and improve quality of life. Moving forward, our findings provide valuable insights for developing holistic approaches to support the mental health of SMA patients and their families.

## Electronic supplementary material

Below is the link to the electronic supplementary material.


Supplementary Material 1



Supplementary Material 2



Supplementary Material 3


## Data Availability

The data that support the findings of this study are available from Dr. M. Flotats-Bastardas but restrictions apply to the availability of these data, which were used under license for the current study, and so are not publicly available. Data are however available from the authors upon reasonable request and with permission of Dr. M. Flotats-Bastardas.

## Abbreviations

ADHD: Attention-deficit/Hyperactivity Disorder
GSI: Global Severity Index
Mini-SCL: Mini-Symptom-Checklist
SCL-90-R: Symptom Checklist
SD: Standard deviation
SDQ: Strengths and Difficulties Questionnaire
SMA: Spinal Muscular Atrophy
SMN1: Survival motor neuron gene
TPBS: Total problem behavior score
TREAT-NMD: Translational Research in Europe for the Assessment and Treatment of Neuromuscular Disease
