# Supplementary material for: Psychological symptoms in individuals with Spinal Muscular Atrophy (SMA) and their caregivers – results from a nation-wide study in Germany
Source: Orphanet J Rare Dis. 2026 Aug 26;21:299. doi: 10.1186/s13023-026-04558-1 (PMC13523259; doi:10.1186/s13023-026-04558-1)
Supplement: Supplementary file 1 [file 13023_2026_4558_MOESM1_ESM.pdf]

Additional file 1: Self- and proxy-reported psychological symptoms of children and their caregivers

	Total	SMA1	SMA2	SMA3[a]	Non-sitters	Sitters	Walkers
**Children with SMA –** *Self-reported SDQ – mean scores (SD)*	**n=7**	**n=1**	**n=4**	**n=2**	**n=2**	**n=3**	**n=2**
Emotional Problems	2.1 (1.5)	1.0	2.5 (1.0)	2.0 (2.8)	3.0 (0.0)	1.7 (1.2)	2.0 (2.8)
Conduct Problems	0.7 (0.8)	2.0	0.3 (0.5)	1.0 (0.0)	0.0 (0.0)	1.0 (1.0)	1.0 (0.0)
Hyperactivity	1.9 (2.1)	2.0	2.0 (2.7)	1.5 (2.1)	0.5 (0.7)	3.0 (2.6)	1.5 (2.1)
Peer Problems	2.0 (1.0)	3.0	2.3 (0.5)	1.0 (1.4)	2.0 (0.0)	2.7 (0.6)	1.0 (1.4)
Prosocial Behavior	8.7 (1.5)	10.0	8.3 (1.7)	9.0 (1.4)	7.5 (2.1)	9.3 (1.2)	9.0 (1.4)
Total score	6.7 (4.0)	8.0	7.0 (4.1)	5.5 (6.4)	5.5 (0.7)	8.3 (4.5)	5.5 (6.4)
**Children with SMA –** *Proxy-reported SDQ – mean scores (SD)*	**n=21**	**n=4**	**n=9**	**n=8**	**n=3**	**n=11**	**n=7**
Emotional Problems	2.1 (2.3)	2.0 (1.4)	2.1 (2.2)	2.3 (2.9)	3.3 (3.1)	2.0 (2.0)	1.9 (2.5)
Conduct Problems	1.3 (1.0)	1.5 (0.6)	1.1 (0.9)	1.4 (1.3)	1.3 (0.6)	1.3 (0.9)	1.3 (1.4)
Hyperactivity	2.0 (2.5)	1.8 (2.4)	1.9 (2.5)	2.1 (2.9)	2.3 (2.3)	1.6 (2.3)	2.3 (3.0)
Peer Problems	1.9 (1.8)	2.5 (2.9)	1.7 (1.7)	1.8 (1.5)	2.3 (2.1)	2.2 (2.0)	1.1 (1.3)
Prosocial Behavior	8.5 (1.2)	9.0 (0.8)	8.4 (1.5)	8.4 (1.2)	8.0 (2.6)	8.9 (0.8)	8.1 (1.1)
Total score	7.2 (5.4)	7.8 (4.3)	6.8 (5.0)	7.5 (6.9)	9.3 (7.5)	7.1 (3.7)	6.6 (7.4)
**Adults with SMA –** *Self-reported Mini-SCL – mean T values (SD)*	**n=82**	**n=3**	**n=28**	**n=51**	**n=7**	**n=55**	**n=20**
Depression	51.7 (8.7)	56.3 (4.6)	51.0 (7.3)	51.8 (9.6)	54.3 (9.5)	51.6 (8.6)	51.3 (9.1)
Anxiety	53.7 (9.7)	47.3 (11.0)	53.1 (8.5)	54.5 (10.3)	52.0 (14.9)	52.9 (9.5)	56.5 (8.1)
Somatization	55.3 (8.7)	58.0 (8.5)	54.4 (8.6)	55.7 (9.0)	55.4 (10.1)	55.1 (8.6)	55.9 (9.2)
Total Score	54.4 (8.7)	54.7 (8.1)	53.4 (7.6)	55.0 (9.4)	55.4 (10.9)	54.0 (8.6)	55.3 (8.7)
**Caregivers –** *Self-reported Mini-SCL – mean T values (SD)*	**n=67**	**n=6**	**n=25**	**n=36**	**n=10**	**n=40**	**n=17**
Depression	53.7 (9.8)	61.0 (7.2)	52.1 (7.9)	53.6 (10.9)	55.3 (6.5)	54.4 (10.5)	51.3 (9.4)
Anxiety	54.8 (9.2)	62.5 (5.0)	52.8 (8.0)	54.9 (10.0)	57.1 (9.9)	54.8 (9.5)	53.5 (8.5)
Somatization	52.5 (9.5)	58.0 (9.9)	50.1 (7.4)	53.2 (10.4)	51.3 (9.6)	52.1 (9.8)	54.2 (8.8)
Total Score	54.3 (9.7)	62.5 (5.6)	51.0 (8.0)	54.6 (9.7)	55.2 (7.9)	54.5 (10.4)	53.4 (9.1)

[a] SMA3 also included one patient with SMA4
