# Supplementary material for: Psychological symptoms in individuals with Spinal Muscular Atrophy (SMA) and their caregivers – results from a nation-wide study in Germany
Source: Orphanet J Rare Dis. 2026 Aug 26;21:299. doi: 10.1186/s13023-026-04558-1 (PMC13523259; doi:10.1186/s13023-026-04558-1)
Supplement: Supplementary file 2 [file 13023_2026_4558_MOESM2_ESM.pdf]

Additional file 2: Spearman correlations of SMA patient and caregiver mental health symptoms

	**Parental/Caregiver psychopathology (Mini-SCL, *T-values*)**			
	Depression	Anxiety	Somatization	Total (GSI)
**Children with SMA –** *Parent-reported SDQ scores (n=21)*				
Emotional problems	.586**			.567**
Conduct problems		.515*		.448*
Hyperactivity				
Peer Problems	.627**			.444*
Prosocial Behavior				
Total	.601**			.653**
**Adults with SMA –** *Self-reported Mini-SCL T-values (n=46)*				
Depression	.443**	.367*		.370*
Anxiety	.470**	.543**	.495**	.528**
Somatization			.314*	
Total	.325*	.450**	.444**	.412**

Only significant correlations are outlined. * = p<.05; ** = p<.01
