# Supplementary material for: Psychological symptoms in individuals with Spinal Muscular Atrophy (SMA) and their caregivers – results from a nation-wide study in Germany
Source: Orphanet J Rare Dis. 2026 Aug 26;21:299. doi: 10.1186/s13023-026-04558-1 (PMC13523259; doi:10.1186/s13023-026-04558-1)
Supplement: Supplementary file 3 [file 13023_2026_4558_MOESM3_ESM.pdf]

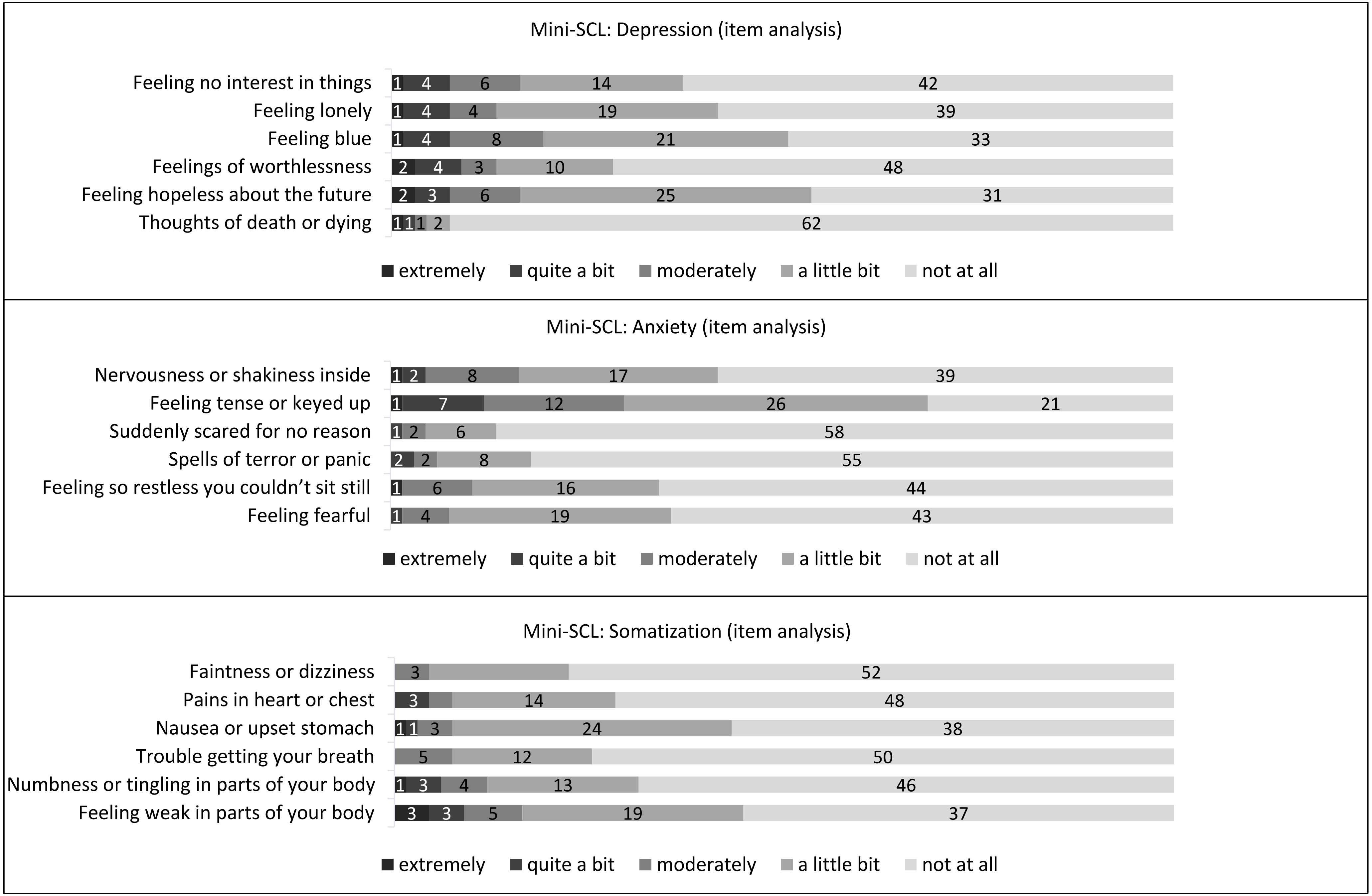

Figure 3: Item analysis of Mini-SCL scales ‚Depression', ‚Anxiety' and ‚Somatization' in caregivers (self-report)
